# Intra- and Interspecies Variability of Single-Cell Innate Fluorescence Signature of Microbial Cell

**DOI:** 10.1128/AEM.00608-19

**Published:** 2019-08-29

**Authors:** Yutaka Yawata, Tatsunori Kiyokawa, Yuhki Kawamura, Tomohiro Hirayama, Kyosuke Takabe, Nobuhiko Nomura

**Affiliations:** a Faculty of Life and Environmental Sciences, University of Tsukuba, Tsukuba, Japan; b Microbiology Research Center for Sustainability, University of Tsukuba, Tsukuba, Japan; c Graduate School of Life and Environmental Sciences, University of Tsukuba, Tsukuba, Japan; d College of Agro-biological Resource Sciences, University of Tsukuba, Tsukuba, Japan; Shanghai Jiao Tong University

**Keywords:** autofluorescence, confocal microscopy, machine learning, microspectroscopy, minimally invasive analysis, single-cell analysis

## Abstract

Here we analyzed the innate fluorescence signature of the single microbial cell, within both clonal and mixed populations of microorganisms. We found that even very similarly shaped cells differ noticeably in their autofluorescence features and that the innate fluorescence signatures change dynamically with growth phases. We demonstrated that machine learning models can be trained with a data set of single-cell innate fluorescence signatures to annotate cells according to their phenotypes and physiological status, for example, distinguishing a wild-type Aspergillus nidulans cell from its nitrogen metabolism mutant counterpart and log-phase cells from stationary-phase cells of Pseudomonas putida. We developed a minimally invasive method (confocal reflection microscopy-assisted single-cell innate fluorescence [CRIF] analysis) to optically extract and catalog the innate cellular fluorescence signatures of each of the individual live microbial cells in a three-dimensional space. This technique represents a step forward from traditional techniques which analyze the innate fluorescence signatures at the population level and necessitate a clonal culture. Since the fluorescence signature is an innate property of a cell, our technique allows the prediction of the types or physiological status of intact and tag-free single cells, within a cell population distributed in a three-dimensional space. Our study presents a blueprint for a streamlined cell analysis where one can directly assess the potential phenotype of each single cell in a heterogenous population by its autofluorescence signature under a microscope, without cell tagging.

**IMPORTANCE** A cell’s innate fluorescence signature is an assemblage of fluorescence signals emitted by diverse biomolecules within a cell. It is known that the innate fluoresce signature reflects various cellular properties and physiological statuses; thus, they can serve as a rich source of information in cell characterization as well as cell identification. However, conventional techniques focus on the analysis of the innate fluorescence signatures at the population level but not at the single-cell level and thus necessitate a clonal culture. In the present study, we developed a technique to analyze the innate fluorescence signature of a single microbial cell. Using this novel method, we found that even very similarly shaped cells differ noticeably in their autofluorescence features, and the innate fluorescence signature changes dynamically with growth phases. We also demonstrated that the different cell types can be classified accurately within a mixed population under a microscope at the resolution of a single cell, depending solely on the innate fluorescence signature information. We suggest that single-cell autofluoresce signature analysis is a promising tool to directly assess the taxonomic or physiological heterogeneity within a microbial population, without cell tagging.

## Abstract (precis)

A cell’s innate fluorescence signature is an assemblage of fluorescence signals emitted by diverse biomolecules within a cell. It is known that the innate fluoresce signature reflects various cellular properties and physiological statuses; thus, they can serve as a rich source of information in cell characterization as well as cell identification. However, conventional techniques focus on the analysis of the innate fluorescence signatures at the population level but not at the single-cell level and thus necessitate a clonal culture. In the present study, we developed a technique to analyze the innate fluorescence signature of a single microbial cell. Using this novel method, we found that even very similarly shaped cells differ noticeably in their autofluorescence features, and the innate fluorescence signature changes dynamically with growth phases. We also demonstrated that the different cell types can be classified accurately within a mixed population under a microscope at the resolution of a single cell, depending solely on the innate fluorescence signature information. We suggest that single-cell autofluoresce signature analysis is a promising tool to directly assess the taxonomic or physiological heterogeneity within a microbial population, without cell tagging.

## INTRODUCTION

A cell’s innate fluorescence signature, an assemblage of autofluorescence signals emitted by diverse biomolecules within the cell (1), is known to reflect various cellular properties and physiological statuses. Previous studies have demonstrated that analysis of fluorescence signatures, for example, when coupled with a principal-component analysis (PCA), allows tag-free analysis of cell types and physiological status within live and intact microbial colonies, bulk microbial culture suspensions (2, 3), active sludges (4), mammalian tissues (5, 6), and mammalian cells (1, 7).

However, innate fluorescence signature analysis at the level of single microbial cells has remained rare, with one notable exception (8), due mainly to the small cell size and the fact that environmental microbial communities are often organized in a three-dimensional (3D) space, for example, by the formation of a biofilm. Here we analyzed single-cell innate fluorescence signatures of microbial cells under a microscope, within both clonal and mixed populations of microorganisms. To this end, we developed a minimally invasive method, which we call confocal reflection microscopy-assisted single-cell innate fluorescence (CRIF) analysis, to optically extract and catalog the innate cellular fluorescence signatures of each of the individual live cells in a three-dimensional space. We combined reflection confocal microscopy (9, 10) and confocal microspectroscopy techniques to achieve reliable extraction of the innate fluorescence signatures from each of the individual cells. Using a range of model organisms, we found that even very similarly shaped cells differ noticeably in their autofluorescence features. Furthermore, we demonstrate that machine learning models can be trained with a single-cell fluorescence signature data set to annotate cells according to their type and physiological status.

## RESULTS

**FIG 1:**
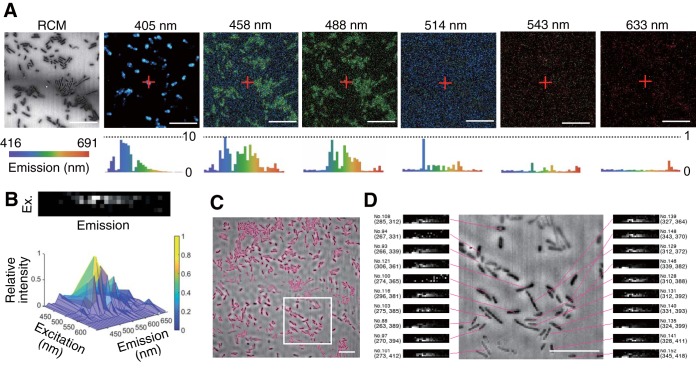
(A) Reflection confocal microscopy (RCM) and confocal microspectroscopy images (labeled by their excitation wavelengths) of P. putida KT2440. Confocal microspectroscopy images are represented as “true-color” images (i.e., the color in the image corresponds to the emission wavelength). Confocal reflection microscopy images are represented as grayscale images that reflect the relative signal intensity. Histograms indicate relative fluorescence intensity spectra in the range of 416 to 691 nm, for the pixel marked by a red cross in the microscopy images. Each bin of the histogram represents a spectral window with a width of 8 nm. Cells appear darker than the background in the confocal reflection microscopy image due to the lower refraction index than for the coverslip. (B) Reconstructed single-cell hyperspectrum presented as a surface plot and as a 2D grayscale image, with six rows for the six excitation wavelengths and a column for each of the 32 bins of the emission spectrum. (C) Visual representation of cell contour recognition by the image analysis routine. A bright-red border indicates the cell contour detected based on intensity gradients. (D) Visual representation of the link between each cell and its single-cell hyperspectrum. The relative *xy* position (pixel counts from the top left corner in a 500- by 500-pixel image) of a cell center of mass and an identification number assigned to each cell are shown beside each hyperspectrum. The images show the 2D projection of z-stack images. Bars, 10 μm.

Figure 1A shows an example data set acquired using our routine for the soil bacterial strain Pseudomonas putida KT2440 (11). In each plane of a z-stack, a reflection confocal image was acquired first, followed by six multichannel confocal microspectroscopy images, in a sequence from longest to shortest excitation wavelength. The innate fluorescence signatures of each of the individual cells (Fig. 1B) were reconstructed by image processing that recognized the contours of each cell (Fig. 1C; see also Fig. S1 in the supplemental material), creating a bundle of six fluorescence spectra (hyperspectrum) linked to the positional information for each cell. Any background fluorescence (Fig. S2) was subtracted from the cell’s hyperspectrum. Figure 1D shows the part of the image in which we assigned the hyperspectrum to one of the 221 cells in the field of view (Fig. S3). The use of a confocal platform allows cellwise averaging to be performed with either a two-dimensional (2D) (Fig. 1D and Fig. S3) or a 3D (Fig. S4) projection of the z-stack data set.

**FIG 2:**
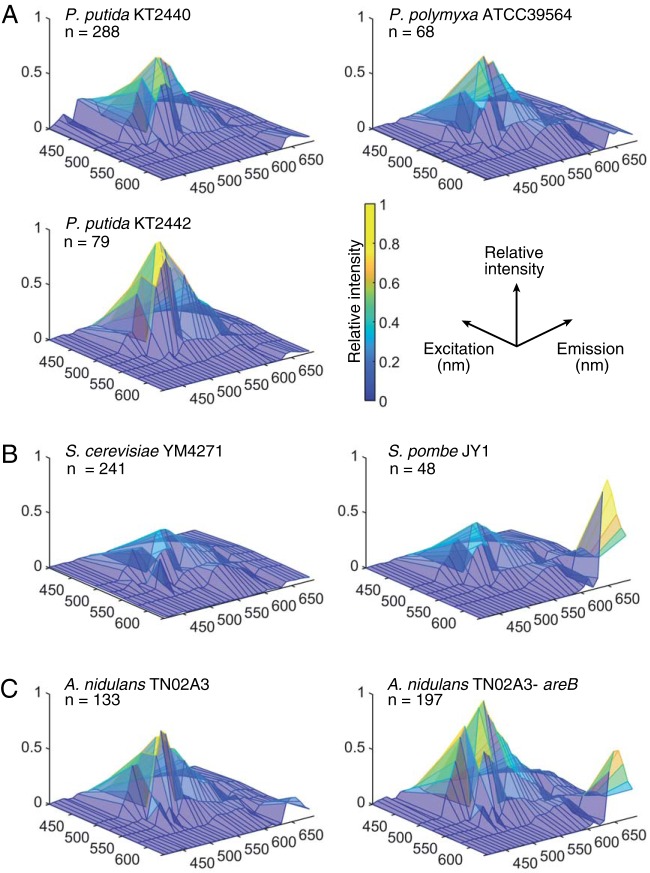
Hyperspectra of bacterial, fungal, and yeast strains. Hyperspectra are presented as surface plots, where *x*, *y*, and *z* axes represent excitation wavelengths, emission wavelengths, and averaged relative fluorescence intensities (color scale), respectively. (A) P. putida KT2440, P. putida KT2442, and *P. polymyxa* ATCC 39564; (B) the budding yeast S. cerevisiae YM4271 and the fission yeast S. pombe JY1; (C) wild-type and nitrogen regulator mutant strains of the filamentous fungus A. nidulans TN02A3. Panels show the hyperspectra averaged over each population of size *n*.

**FIG 3:**
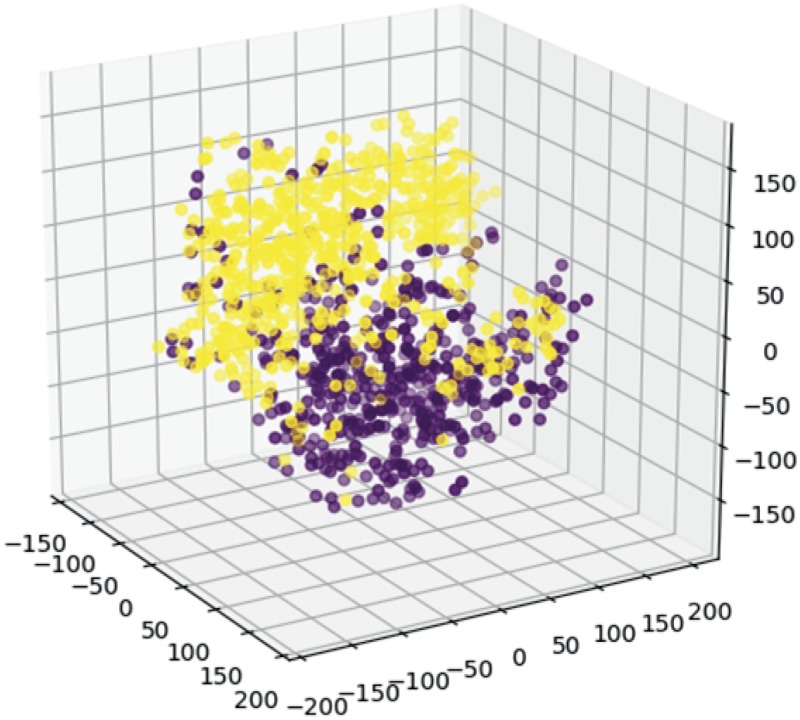
Variance of two hyperspectrum matrix pairs of *P. polymyxa* (purple) (*n* = 491) and P. putida (yellow) (*n* = 607) visualized using the t-distributed stochastic neighbor embedding (t-SNE) method.

The fluorescence signatures differed among 7 strains compared in this study. We extracted innate fluorescence signatures from cell populations of bacterial, fungal, and yeast strains. While minor within-population variability was observed for each population (Fig. S5 and Movie S1), the population-averaged fluorescence signatures of the populations differed noticeably (Fig. 2 shows the fluorescence signature averaged over a population). To further resolve this interspecies variability, we performed PCA and t-distributed stochastic neighbor embedding (t-SNE) analyses of taxonomically close strain pairs. Distinct cluster formation upon t-SNE analysis and PCA (Fig. 3 and Fig. S6) was observed between two soil bacterial species (Paenibacillus polymyxa ATCC 39564 and P. putida KT2440) as well as between wild-type (KT2440) and rifampin-resistant derivative (KT2442) (12) strains of P. putida. Distinct cluster formations (Fig. S6) were also observed between budding yeast (Saccharomyces cerevisiae YM4271) (13) and the fission yeast Schizosaccharomyces pombe JY1 (14) as well as between the wild type and a nitrogen regulator deletion mutant of the filamentous fungus Aspergillus nidulans TN02A3 (15). These results indicate that the single-cell innate fluorescence signature can vary considerably even among clonal populations. Our results also suggest that even under the given same ambient conditions, different species or strains can generate considerably different innate fluorescence signatures.

**FIG 4:**
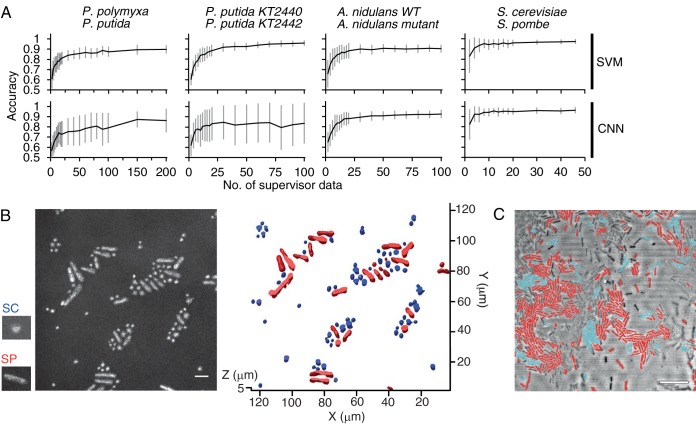
(A) Two-class classification accuracies of the SVM (top row) and the CNN (bottom row) models with various numbers of supervisor data. The *y* axes denote classification accuracy between *P. polymyxa* ATCC 39564 and P. putida KT2440, P. putida KT2440 and P. putida KT2442, wild-type (WT) and nitrogen regulator mutant strains of the filamentous fungus A. nidulans TN02A3, and the budding yeast S. cerevisiae YM4271 and the fission yeast S. pombe JY1. Data show average accuracies of 100 models trained independently, with bars representing the standard deviations. (B) Superposition of virtual labels (blue, S. cerevisiae; red, S. pombe) on a mixed population of S. cerevisiae and S. pombe JY1. The left black-and-white panel shows a maximum-intensity projection image calculated from the z-stack of reflection confocal images, and small panels show typical morphologies of S. cerevisiae (SC) and S. pombe JY1 (SP). (C) Superimposition of virtual labels (blue, *P. polymyxa*; red, P. putida) on a mixed population of *P. polymyxa* ATCC 39564 and P. putida KT2440. Note that a certain portion of the cell population was not recognized by the image processing algorithm that detected the signal intensity gradient in a reflection confocal microscopy image. Virtual labels are generated based on classification by the SVM model pretrained with 100 supervisor data for each species, which is generated with an isolated (nonmixed) population. The image shows a 3D (B) or 2D (C) projection of z-stack confocal reflection microscopy images. Bars, 10 μm.

Machine learning models were trained by single-cell innate fluorescence signatures to predict cell types. In most cases, for both the support vector machine (SVM) and the convolutional neural network (CNN), the accuracy of two-class classification approached or exceeded 90% with a relatively small number (<100) of supervisor data (Fig. 4A), corroborating the distinct clusters in the PCAs (Fig. S6). Furthermore, we applied the trained models to predict cell types and annotate intact cells distributed in a three-dimensional space, relying only on the fluorescence signature information. Figure 4B shows the result of cell-by-cell classification superimposed on the reconstructed confocal microscopy image of a mixed population of S. cerevisiae YM4271 and S. pombe JY1. The innate fluorescence signature-based annotation matched the morphological characteristics of the two species (S. pombe cells are larger and more elongated than spherical S. cerevisiae cells) at an accuracy of 94.3% (standard deviation [SD] = 5.1 [triplicate experiments]). Figure 4C shows an example of the predictive annotation, where we applied the SVM model trained to >90% accuracy (Fig. 4A) in cell type prediction, for a mixed population of P. polymyxa ATCC 39564 and P. putida KT2440.

**FIG 5:**
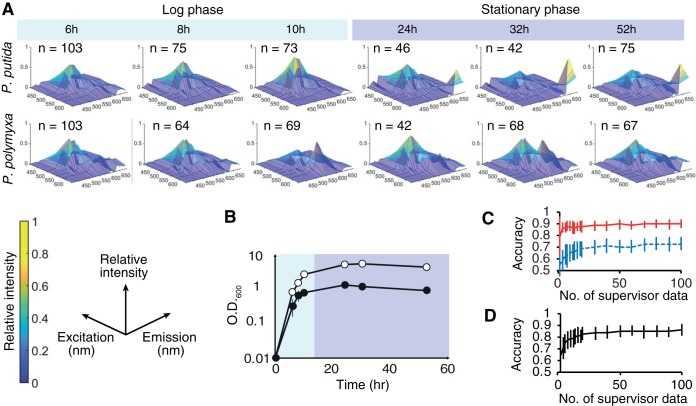
(A) Hyperspectra of P. putida KT2440 and *P. polymyxa* ATCC 39564 at various growth stages. Panels show the hyperspectra averaged over each population of size *n*. (B) Time course of the optical density at 600 nm (O.D._600_) for *P. polymyxa* ATCC 39564 (open circles) and P. putida KT2440 (filled circles). Growth data are the averages of results from triplicate experiments. (C) Classification accuracy between the log and stationary phases of *P. polymyxa* ATCC 39564 (blue) and P. putida KT2440 (red) cells by the SVM with various numbers of supervisor data. (D) Two-class classification accuracy for *P. polymyxa* ATCC 39564 and P. putida KT2440 cells by the SVM with various numbers of supervisor data, with all growth stages included. Bars show the standard deviations.

The single-cell innate fluorescence signature also reflected the physiological status of cells, specifically the growth stages. The innate fluorescence signatures of *P. polymyxa* and P. putida changed over time (Fig. 5A), in parallel with the growth phase (Fig. 5B), while the morphology of the cells remained largely unchanged. Table 1 shows the confusion matrix of the SVM model in the six-class classification among different growth stages within each of the two soil bacteria. The SVM model consistently predicted the correct growth stage with highest probability (with accuracy in the range of 0.43 to 0.87) for both soil bacteria. Closer inspection revealed differences in the temporal dynamics of cell physiology between the two soil bacteria. In P. putida, the fluorescence signatures showed a biphasic change between stationary and log growth phases, with stationary-phase fluorescence signatures being commonly characterized by a strong long-wavelength emission peak, and the fluorescence signatures were rather similar within each growth phase. In *P. polymyxa*, in contrast, the innate fluorescence signatures constantly fluctuated throughout the culture period. These differential temporal dynamics were reflected in the classification result, where the SVM model could accurately distinguish log-phase cells from stationary-phase cells for P. putida but not *P. polymyxa* (Fig. 5C). Intriguingly, the SVM model trained by the data set that includes all of the growth stages (6, 8, 10, 24, 30, and 52 h) classified the two species at an accuracy of approximately 90%, regardless of the growth stage of the test data (Fig. 5D). These results suggest that the machine learning models can be trained to classify two populations of bacteria, even in the case where each population includes cells of various physiological statuses. Taken together, these results indicate that single-cell innate fluorescence is a transient signature and reflects dynamics of cellular physiology that is unique to a cell type.

**TABLE 1:** Confusion matrices of growth stage prediction by the SVM models

Model	No. of instances of indicated growth stage^*a*^					
	6 h	8 h	10 h	24 h	30 h	52 h
P. putida						
Prediction						
6 h	17	5	2	1	0	0
8 h	5	15	6	0	0	0
10 h	5	1	14	1	0	0
24 h	3	0	0	6	2	2
30 h	0	0	0	5	8	1
52 h	1	2	0	1	3	20
Accuracy	0.55	0.65	0.64	0.43	0.62	0.87

*P. polymyxa*						
Prediction						
6 h	23	2	3	0	3	3
8 h	2	14	3	0	2	0
10 h	2	2	62	2	4	3
24 h	1	0	2	9	1	0
30 h	2	0	8	1	10	2
52 h	1	1	2	1	1	8
Accuracy	0.74	0.74	0.78	0.69	0.48	0.50

a Shading denotes correct identifications.

## DISCUSSION

The fluorescence signature of a whole microbial colony or a bulk culture suspension, the focus of traditional microbial autofluorescence research, is inherently an averaged mixture of signals from a large number of cells as well as noncell signals of medium components, secreted metabolites, and extracellular matrices. Our use of reflection confocal microscopy, which provides an independent source of information to identify cell contours, provides an important advance by allowing the selective extraction of fluorescence signals from individual cells (see Fig. S1 in the supplemental material), distributed in a three-dimensional space (Fig. S4).

Techniques to determine cell types or physiological status, such as DNA or cell content extraction (16, 17), fluorescence *in situ* hybridization (FISH) (18), and the introduction of fluorescent reporter genes into a genome, commonly require invasive tagging or manipulation of the cells. In contrast, the fluorescence signatures that we exploit are innate properties of the cells, and hence, our technique allows the predictive annotation of cellular phenotype (Fig. 4) or physiological status (Fig. 5) of intact cells, which is not constrained by the availability of genetic tools. The fact that this spatial mapping can be achieved in a tag-free and noninvasive fashion implies that it can be applied to the resolution of the temporal development of cell distribution and physiological state, an ideal tool to analyze phenotypic heterogeneity within a cell population. Another potential application would be streamlined screening, where one can directly assess the potential phenotype of each candidate by their autofluorescence signature without cell tagging, even prior to clonal culture.

We acknowledge that there are some limitations in this study. First, although vitamins (e.g., flavin), coenzymes (e.g., NADH), and lipofuscin pigments are suggested to be major sources of cellular autofluorescence (4), we have not analyzed which intracellular molecules characterize the innate fluorescence signatures that distinguish cell types. However, our results demonstrate the effectiveness of innate fluorescence signature analysis as a tool for predicting cell types and physiological status, independent of precise knowledge on how intracellular chemical compositions are mapped onto innate fluorescence signatures. A combined analysis with single-cell metabolomics would help to resolve the chemical nature of the peaks found in the innate fluorescence signatures. Second, while our results demonstrated that CRIF can reveal intra- and interspecies variabilities in innate fluorescence signatures, we have not yet systematically or exhaustively explored such variabilities (e.g., whether the difference between genera is consistently greater than that between species), which is certainly an attractive avenue. Nevertheless, we believe that the present study provides a technological breakthrough necessary for such exciting new explorations.

We recognize a few noteworthy trade-offs compared to more traditional methods. First, in the current configuration, CRIF requires a confocal microscope with spectral resolution, certainly a considerable investment compared to a simple fluorescence microscope used for FISH and other fluorescence protein tagging techniques. Second, for predictive annotation, the classification model requires innate fluorescence signatures sampled under a range of conditions, to confer robustness against environmental variables, although we have demonstrated that constructing such a robust classification model is possible (Fig. 5D). This need for a robustly pretrained classification model, stemming from the fact that an innate fluorescence signature reflects the physiological state or “the instantaneous phenotype” of a cell (Fig. 5A), suggests that well-characterized species/strains are suitable targets for the predictive annotation technique. Other applications of CRIF, on the other hand, would depend less on, and are not necessarily constrained by, such prior knowledge. For example, analysis of the phenotypic heterogeneity within a clonal microbial population by dimensionality reduction (Fig. 3 and Fig. S5 and S6) does not require a pretrained classification model.

Analysis of both cell morphology (19) and innate fluorescence signatures allows us to infer cellular taxonomy (Fig. 4) and physiological state (Fig. 5), and both sources can be tapped in a minimally invasive fashion. In regard to this point, we suggest that the innate fluorescence signature is, for characterization of a microorganism, as important as its morphology. The technique to isolate, recognize, and track the innate fluorescence signatures of each of the individual cells in three-dimensional space developed in this study will bring about a unique opportunity to probe into the dynamics of heterogenous microbial populations, all in a minimally invasive and tag-free fashion.

## MATERIALS AND METHODS

### Strains and culture conditions.

The bacterial and fungal strains (*Pseudomonas*, *Paenibacillus*, *Aspergillus*, *Saccharomyces*, and *Schizosaccharomyces*) used in this study are listed in Table 2. For routine culture, *Pseudomonas* and *Paenibacillus* cells were grown in liquid LB medium or on LB agar plates at 30°C. Aspergillus nidulans wild-type and mutant strains were cultured in supplemented minimal medium at 28°C overnight in chambered cover glasses (20). Yeast strains were grown in yeast extract-peptone-dextrose (YPD) medium (Sigma-Aldrich, St. Louis, MO, USA) or on YPD agar (Sigma) plates at 30°C. An orbital shaker (600 rpm) was used for liquid cultures.

**TABLE 2:** Microbial strains used in this study

Species	Strain	Description	Reference
Pseudomonas putida	KT2440	Soil bacterium	11
	KT2442	Rifampicin-resistant variant of KT2440	12

Paenibacillus polymyxa	ATCC 39564	Obtained from the ATCC	

Aspergillus nidulans	TN02A3	Wild type	15
	Δ*areB*	Nitrogen regulator deletion mutant of TN02A3	15

Saccharomyces cerevisiae	YM4271	Obtained from the ATCC	13

Schizosaccharomyces pombe	JY1	Wild type	14

### Experimental setup.

A 1-mm-thick 0.8% (wt/vol) agarose slab placed on a glass slide was used to hold cells for routine confocal scanning microscopy imaging, in order to maintain cells under wet conditions. The agarose slab was placed in a well of a silicone gasket (1 mm thick), and a 1-ml aliquot of the cell suspension was placed on the agarose slab and then gently covered by a glass coverslip. For imaging, we used an upright confocal laser scanning microscope (LSM 880; Carl Zeiss, Oberkochen, Germany) equipped with a 63×, 1.4-numerical-aperture (NA) plan apochromat objective, differential grating, and 32 descanned spectral channels with a GaAsP photoelectron multiplier tube (PMT) array. For reflection confocal microscopy (9, 10), cells were illuminated with a 514-nm laser, and the scattered light was collected through a half-reflection mirror (NT 80/20) and a 1-Airy-unit (AU) pinhole. For multichannel confocal microspectroscopy, cells were illuminated with one of six laser lines (405, 458, 488, 514, 543, and 633 nm), and the emission was collected through a dichroic mirror and a 1-AU pinhole. The voxel sizes were 0.264 by 0.264 by 0.674 μm and 0.264 by 0.264 by 0.871 μm (*x* by *y* by *z*) for confocal reflection microscopy and confocal microspectroscopy, respectively. MBSInVis405, MBS458, MBS488, MBS458/514, MBS488/543, and MBS488/543/633 beam splitters (Carl Zeiss) were used for 405-, 458-, 488-, 514-, 543-, and 633-nm excitation, respectively. The emission within the range of 416 to 691 nm was binned into 32 spectral channels, with each channel having a spectral width of 8 nm. The illumination intensity for each excitation wavelength was measured with a laser power meter and adjusted to 50 μW under the 63× objective. The pixel dwell times were 1.03 μs and 2.06 μs for confocal reflection microscopy and confocal microspectroscopy, respectively.

### Reconstruction of single-cell fluorescence signatures.

A custom MATLAB (MathWorks, Natick, MA, USA) routine was used to reconstruct a hyperspectrum, which has the illumination and the emission wavelengths as axes, for each of the individual cells. The hyperspectrum, the visual representation of innate fluorescence signatures, is linked to each cell’s three-dimensional positional information. Each cell is defined using reflection confocal microscopy, which often excels in the definition of morphological information compared to fluorescence confocal microscopy, particularly when fluorescent signals are weak (see Fig. S1 in the supplemental material). For relatively small cells (e.g., bacterial cells) distributed on a 2D plane, each cell region in the reflection confocal image was segmented and cataloged by determining their outline in a maximum-intensity projection image calculated from the z-stack of the reflection confocal images using a 2D intensity gradient method. For larger cells (e.g., yeast) or cell populations distributed three dimensionally, the cell boundary surfaces were directory determined with 3D volume data. We then used each of the cataloged cell regions as a mask to calculate the signal intensity averaged over the corresponding cell regions in each of the six multichannel confocal microspectroscopy images, thereby obtaining six emission spectra for each cell. This operation thus creates a bundle of six fluorescence spectra (hyperspectrum) linked to the positional information for each cell. For machine learning purposes, a Laplacian filter function (MATLAB) was applied to the 6-by-32 hyperspectrum matrices. To account for any background fluorescence deriving from medium components or the experimental setup (e.g., agarose and coverslip), the hyperspectra of the noncell regions were also generated and averaged over the area (2D) or space (3D), which were then subtracted from the hyperspectrum of the cells. Figure S2 shows the typical background fluorescence in our experimental setups.

### Classification using machine learning models.

We employed principal-component analysis (PCA) and the t-distributed stochastic neighbor embedding (t-SNE) method (21), which has been widely used to reduce dimensions of multidimensional data, to visualize the variance of hyperspectra within a cell population. A support vector machine (SVM) model (22) and a convolutional neural network (CNN) model (23) running in the Python language were used to classify the different types of cells. The SVM and CNN models were constructed and trained using the scikit-learn package and the Chainer package (https://chainer.org/), respectively. For the CNN model, we constructed and trained a four-layer CNN, consisting of two convolutional layers and two linear layers. For both training and classification with the SVM, we generated a 192-dimensional cellular fluorescence intensity vector from the six fluorescence spectra (each made up of 32 spectral channels) linked to each cell. For the CNN, a 6-by-32 hyperspectrum matrix was generated out of the six fluorescence spectra associated with each cell and used as the input to the first convolutional layer. The classification models were trained using a varying number (in the range of 2 to 200) of fluorescence intensity vectors or hyperspectra randomly chosen from the population. The CNN model was trained over 100 epochs, with each epoch consisting of 100 minibatch training cycles. The classification accuracy was evaluated using 50 fluorescence intensity vectors randomly chosen from the population, excluding those that were used for training.

## Supplementary Material

Supplemental file 1

Supplemental file 2
