# Supplementary material for: Intra- and Interspecies Variability of Single-Cell Innate Fluorescence Signature of Microbial Cell
Source: Appl Environ Microbiol. 2019 Aug 29;85(18):e00608-19. doi: 10.1128/AEM.00608-19 (PMC6715841; doi:10.1128/AEM.00608-19)
Supplement: Supplemental file 1 [file AEM.00608-19-s0001.pdf]

Supplementary figures

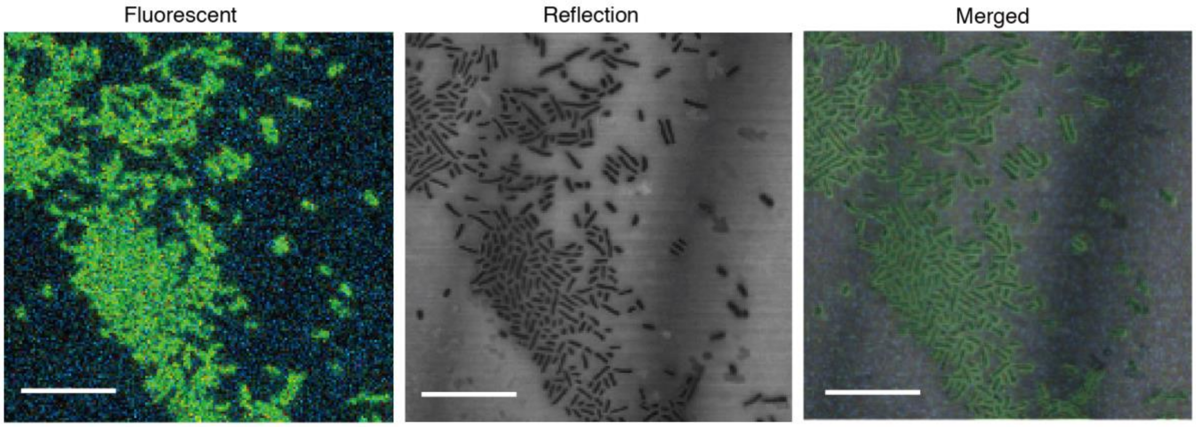

Figure S1. Representative confocal microspectroscopy (*Left*), and reflection confocal microscopy (Middle) images of *P. putida* KT2440. The confocal microspectroscopy image shows the innate fluorescence taken with 458 nm excitation wavelength. The rightmost panel shows a merged image. Scale bars = 10 μm.

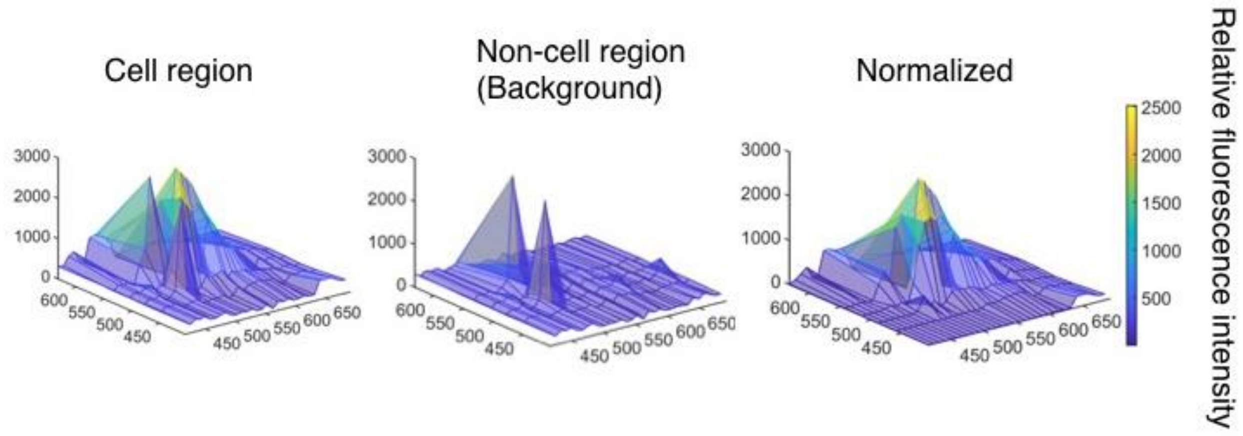

Figure S2. Hyper-spectrums of cells, non-cell region of the image and normalized hyper spectrum. The hyper-spectrum of the non-cell region was averaged over the 2D area. The normalized hyper-spectrum of cells (right) was generated by subtracting the non-cell region hyper spectrum (middle) from the cell region hyper spectrum (left). Hyper-spectrums are presented as surface plots, where X, Y, and Z axes represent excitation wavelengths, emission wavelengths and averaged relative fluorescence intensity (color scale), respectively.

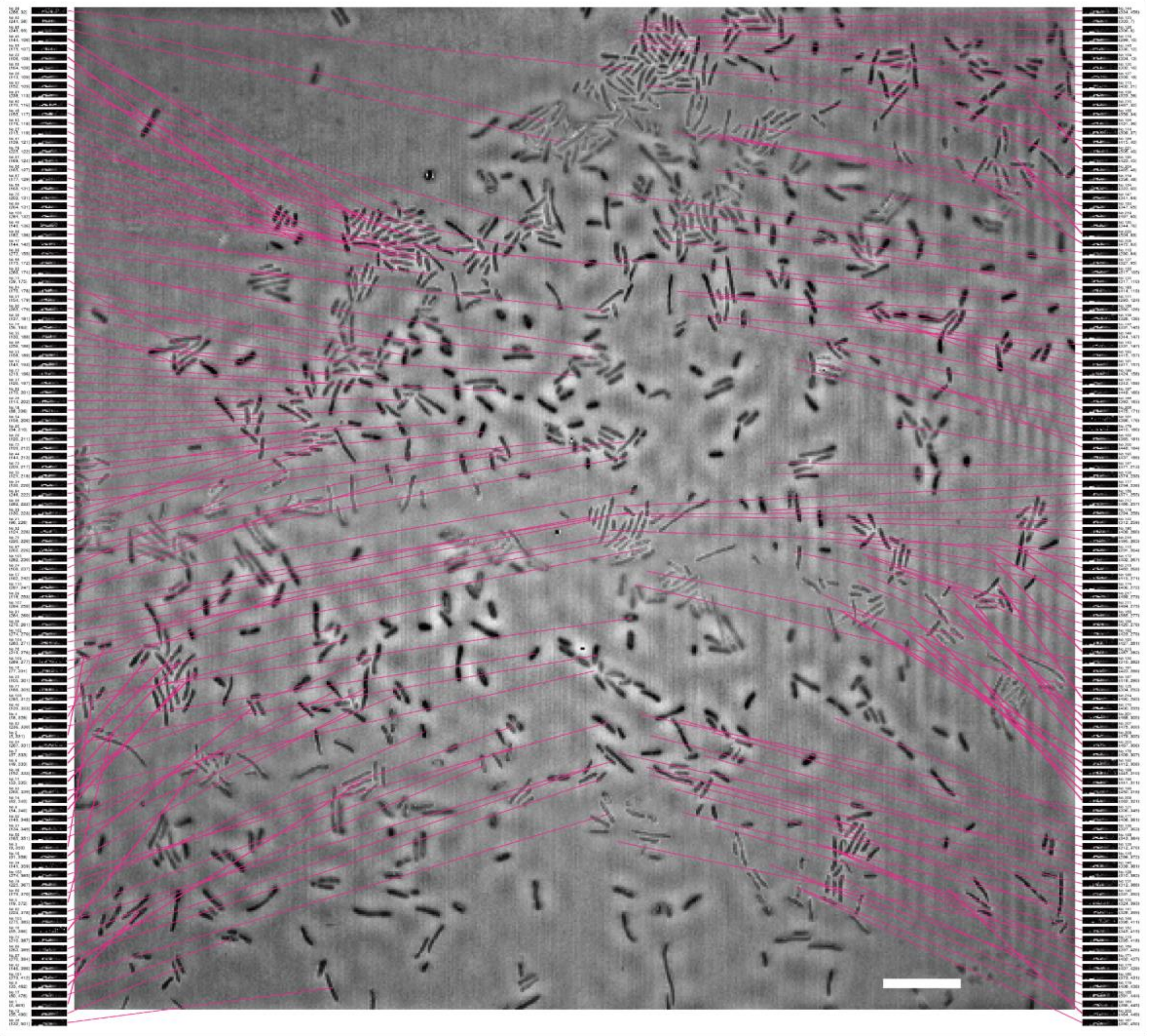

Figure S3. Visual representation of the links between each of *P. putida* KT2440 cells and their single-cell hyper-spectrums. The relative X, Y position and the identification number assigned to each cell is shown next to each hyper-spectrum. Scale bar = 10μm.

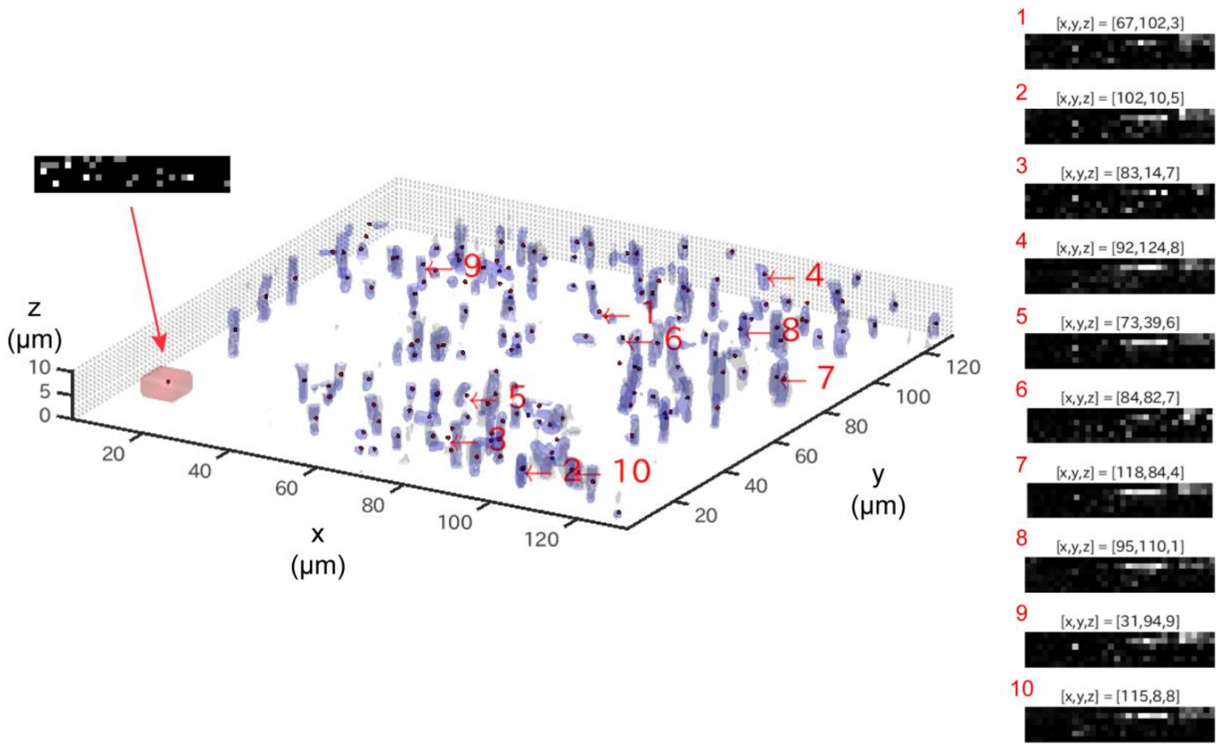

Figure S4. Representative three-dimensional projection image of a *S. cerevisiae* population acquired with the reflection confocal microscopy. Purple shadings represent the cell volumes and red spheres indicate the cell centers of masses. The single-cell hyper-spectrums of the randomly chosen cells are shown as insets. The example background hyper-spectrum of the red shaded portion is shown as inset (left). The X, Y, Z position (μm) of the center of mass and the identification number assigned to each cell are shown above each hyper-spectrum.

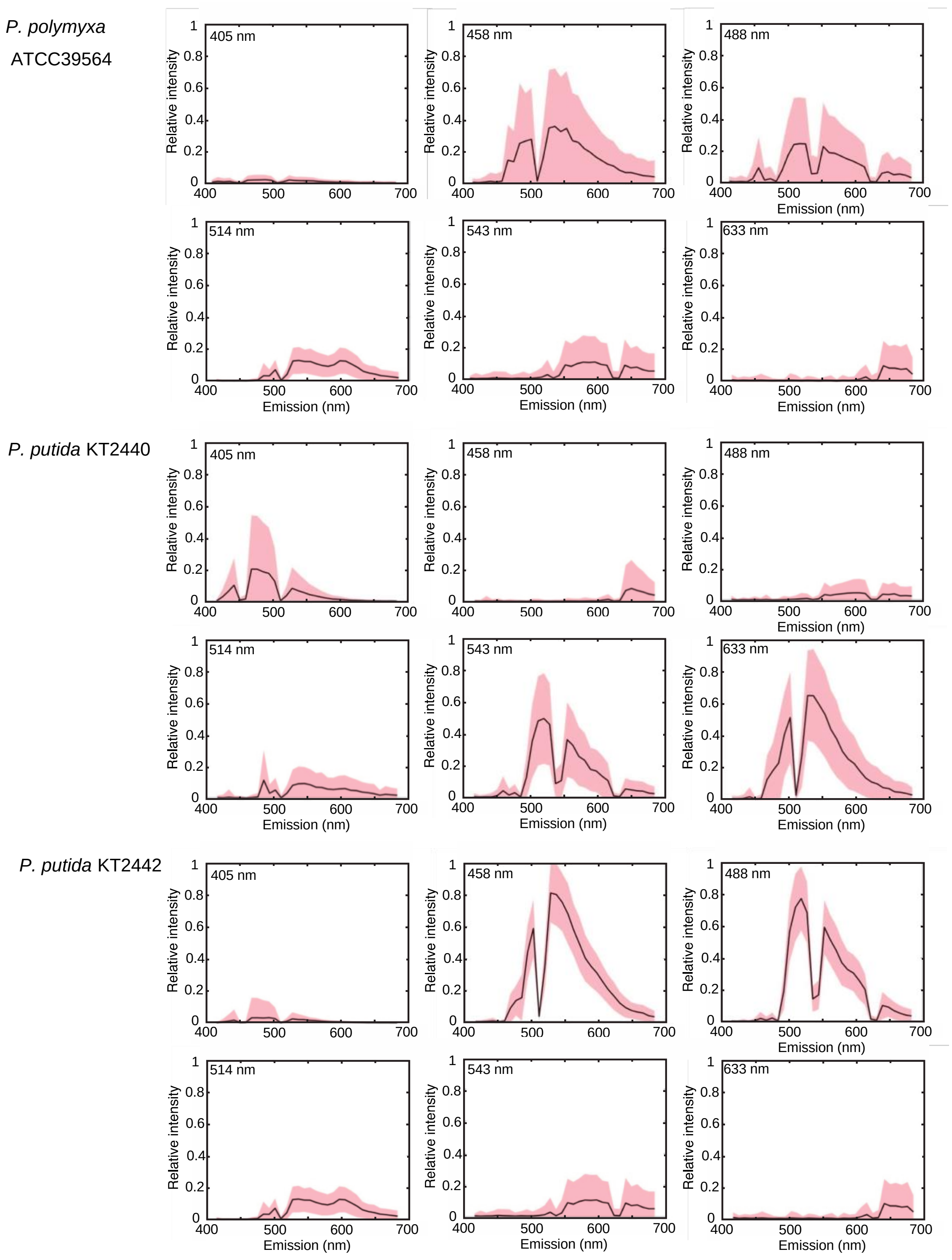

Figure S5 (continues to the next page)

S. cerevisiae YM2471

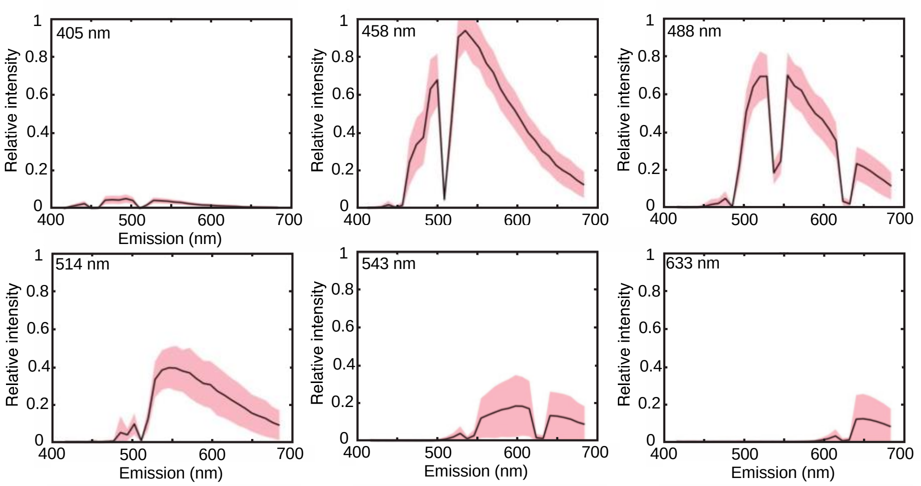

S. pombe JY1

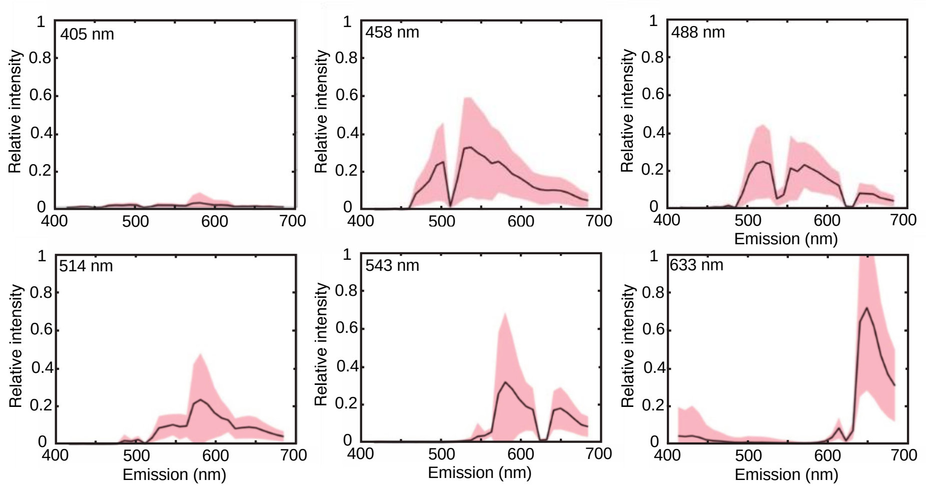

Figure S5 (continues to the next page)

A. nidulans WT

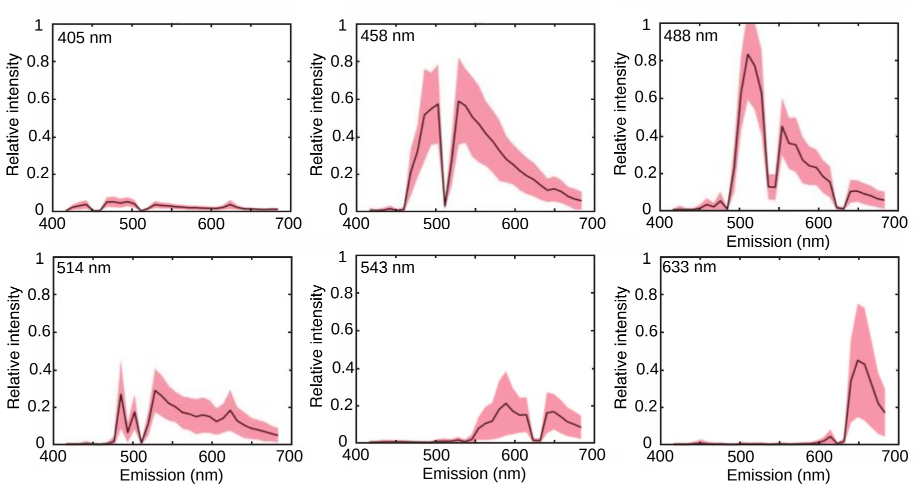

A. nidulans Δ*areB*

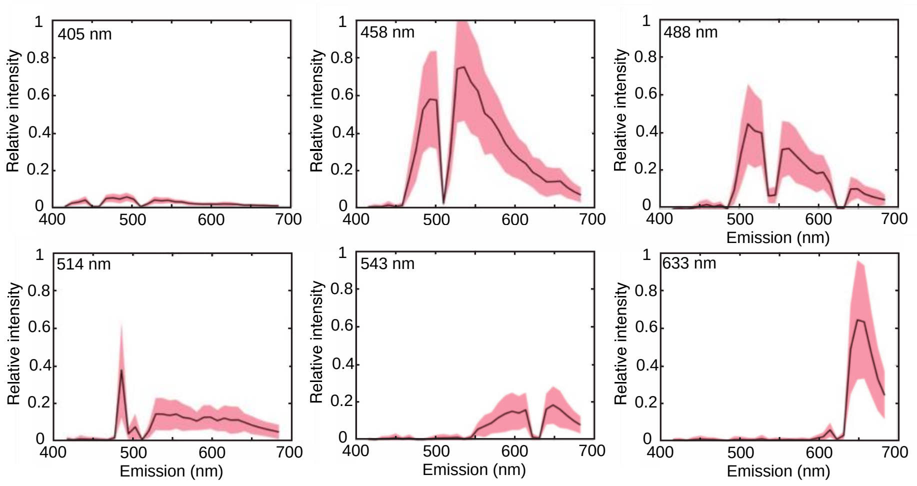

Figure S5. Spectrums of bacteria, fungi, and yeast strains examined in this study (From the top: *P. putida* KT2440, *P. putida* KT2442, *P. polymyxa* ATCC39564, budding yeast *S. cerevisiae* YM4271, fission yeast *S. pombe* JY1, filamentous fungi *A. nidulans* WT, *A. nidulans ΔareB*). Each panel shows a spectrum acquired with each of the excitation wavelengths (shown at upper left in each panel), and X and Y axes represent emission wavelengths and averaged relative fluorescence intensity, respectively. Each spectrum was averaged over the sample size of each population and the red shaded area shows the standard deviation of each spectrum.

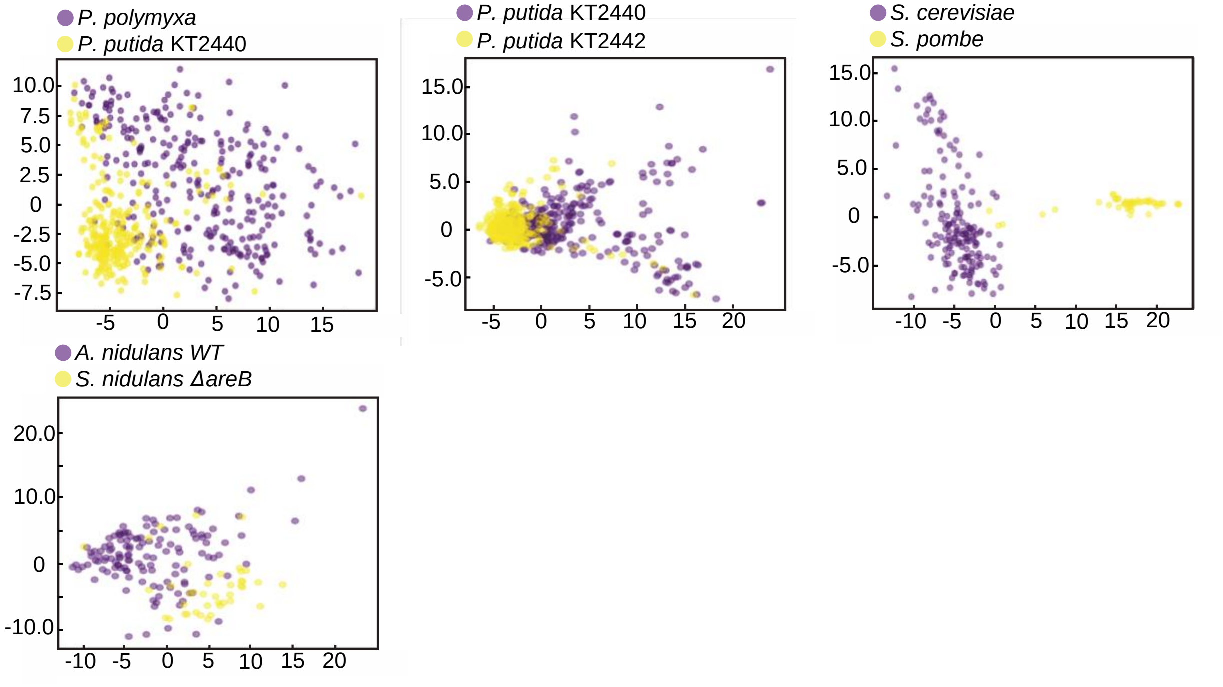

Figure S6. Variance of two hyper-spectrum matrix pairs visualized by PCA. X and Y axes represent PC1 and PC2, respectively. The panels show (from the top) a pair of *P. polymyxa* ATCC39564 (purple, n = 294) and *P. putida* KT2440 (yellow, n = 289), *P. putida* KT2440 (purple, n = 289) and *P. putida* KT2442 (yellow, n = 373), budding yeast *S. cerevisiae* YM4271 (purple, n = 171) and fission yeast *S. pombe* JY1 (yellow, n = 48), and filamentous fungi *A. nidulans* WT (purple, n = 133) and *A. nidulans ΔareB* (yellow, n = 40).

Movie S1. Movie of reconstructed single-cell hyper-spectrum presented as surface plots. Each frame of the movie represents single-cell innate fluorescence signature of each cell in the *P. putida* population shown in Fig. S3.
